# The acuity of paediatric asthma in the emergency department worsened after the COVID-19 pandemic in Alberta, Canada

**DOI:** 10.1186/s13223-026-01045-x

**Published:** 2026-07-06

**Authors:** Behzad Heibati, Amanpreet Gill, Jalal Moolji, Adil Adatia

**Affiliations:** 1 Division of Pulmonary Medicine, Department of Medicine, University of Alberta, Edmonton, AB Canada; 2 Department of Pediatrics, Faculty of Medicine, University of Alberta, Edmonton, AB Canada

**Keywords:** COVID-19, Emergency department, Asthma, Paediatrics, Healthcare utilisation

## Abstract

**Background:**

Asthma affects about 13% of Canadian children and is a common reason for emergency department (ED) visits or hospitalization. The COVID-19 pandemic significantly impacted the health care utilisation of children with asthma. The aim of this study was to investigate how paediatric ED visits were influenced by the pandemic.

**Methods:**

We conducted a retrospective study examining asthma ED visits, admission rates, and presentation acuity for children 2 to 17-years-old from 2011 to 2024. Presentation acuity was determined using Canadian Triage and Acuity Scale (CTAS). Data were collected from the Government of Alberta Interactive Health Data Application and the Alberta Health Services aggregated administrative database. Data were analysed graphically. Interrupted time series (ITS) analysis of monthly ED visits and admissions, with March 2020 as the intervention point.

**Results:**

The mean daily incidence of ED visits remained at 5–10 per million per year for ages 2–12 years, and under 5 per million per year for ages 13–17 years, except for a transient interruption in the early pandemic, in 2020. Following this interruption, the pre-existing trend to worsening acuity and greater rates of hospitalization accelerated in the late pandemic. The acuity and hospitalization rates have remained elevated in the post-pandemic years. Emergency medical services (EMS) visits increased substantially across paediatric subgroups in 2024, ranging from 1.53× to 2.26× higher among children aged 2–12 years compared with pre-pandemic levels. ITS analysis showed a significant immediate decrease in ED visits and admissions, followed by a positive post-intervention slope, indicating gradual recovery over time.

**Conclusion:**

Pre-existing trends in paediatric asthma exacerbations worsened in the years following 2020. Although the incidence of ED visits did not substantially increase, children were more likely to have severe exacerbations and require hospitalization in the late pandemic and post-pandemic years relative to pre-pandemic years.

**Supplementary Information:**

The online version contains supplementary material available at https://doi.org/10.1186/s13223-026-01045-x.

## Background

Asthma is the most common chronic disease in children with a prevalence of ~ 15% globally and in Canada [1, 2]. Asthma exacerbations are a common indication for paediatric Emergency Department (ED) presentation and are a leading cause of hospitalization [1, 2], resulting in a significant economic and humanistic burden in Canada [3, 4].

The epidemiology of paediatric asthma exacerbations, particularly in the post-COVID-19 pandemic period, is incompletely understood. Our group previously reported a gradual decline in ED visits for asthma in adults in the province of Alberta from 2010 to 22, with a steep temporary reduction in 2020 associated with pandemic containment measures, but those presenting to EDs had increased acuity and risk of hospitalization. We speculated that the prevalence of poorly-controlled asthma increased during this period, but patients were hesitant to attend EDs due to overcrowding, unless their symptoms were particularly severe [5].

Subsequently, Lukac et al. described how hospitalizations for acute respiratory diseases in children were affected by the COVID-19 pandemic in Alberta [6].They concluded that the effects of the social restrictions of the early pandemic had ended and typical seasonal trends in respiratory diseases had resumed. Similar findings were obtained from the province of Quebec, where hospitalizations for respiratory viruses sharply dropped in 2020 and returned to pre-pandemic levels by 2022, likely driven by respiratory syncytial virus infection [7]. Rebounds in paediatric hospitalizations for asthma and viral illnesses were also seen internationally, for example in France and Japan [8, 9].

In the present study, we evaluated if there were changes in ED presentation acuity for children with asthma exacerbations in the post-pandemic period.

## Methods

### Study design and data source

We conducted a retrospective, descriptive study examining asthma ED visits, admission rates, and presentation acuity for children 2 to 17-years-old from 2011 to 2024. The data for this study were extracted retrospectively from the Alberta Health Services aggregated administrative database. Data were collected on the number of paediatric asthma ED visits, asthma admissions, and total population size for males and females aged 2–17 years from January 1, 2011 to December 31, 2024. The pandemic period was defined as March 2020 to September 2022 to capture both the initial restriction phase and the subsequent period of ongoing healthcare disruption and recovery.

Acuity of each visit was determined using the Canadian Triage and Acuity Scale (CTAS) score, which is a standardized assessment tool which has been in use in Canadian ED since 1999 for triage and patient flow analysis. The scale ranges from 1 to 5, with 1 being the highest acuity and 5 being the lowest acuity. CTAS data were collected for males and females aged 2–17 years from 2011 to 24. The use of emergency medical services to attend to the ED (both ground and air transport) was also collected as a binary variable. The data were publicly available and research ethics approval was not required. The authors did not have access to information that could identify individual participants during or after data collection.

### Data analysis

Data analysis was performed to assess ED visit incidence, hospital admission rate, ED visit acuity and EMS use, and their trends over time. Data were stratified by sex and the age ranges 2–5 years, 6–12 years, and 13–17 years. These age groups were chosen to align with clinically meaningful developmental stages in paediatric asthma. Children younger than 2 years were excluded due to the difficulty of reliably differentiating asthma from other respiratory conditions in this age group. The mean number of total daily ED visits and ED visits using EMS were calculated per 1 million persons from 2011 to 24. The rate of hospital admission specific to asthma-related diagnoses was calculated by dividing the number of admissions per day by the number of ED visits per day and averaging over each year, in each subgroup. Area graphs were created for each CTAS score for each age category. Seasonal trends in ED visit volume were assessed by constructing heatmaps showing the proportion of the annual total asthma ED visits that occurred in each month. Trends were assessed graphically for each dataset. In addition, we conducted an interrupted time series (ITS) analysis to evaluate changes in ED visits and hospital admissions before, during, and after the COVID-19 pandemic. The data analysis was conducted using R version 4.3.2.

## Results

### Trends in ED visits

**Fig. 1:**
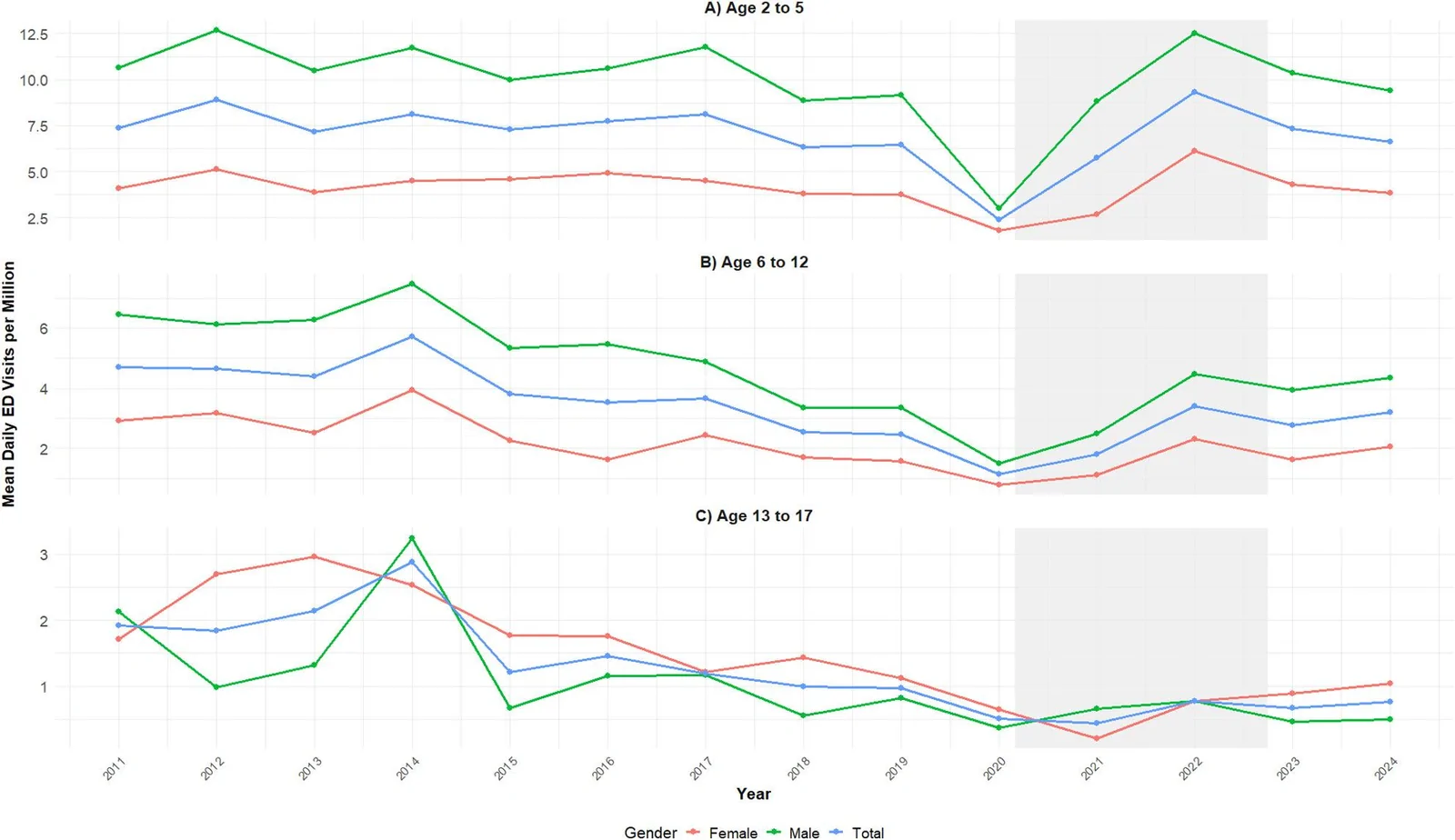
Mean daily ED visits per million across three age groups: 2 to 5 years (**A**), 6 to 12 years (**B**), and 13 to 17 years (**C**), categorized by sex. The grey, shaded area represents the COVID-19 pandemic (Mar 2020-Sep 2022). The values are normalized to census data for the number of Albertan children in each age group

From 2011 to 24 there were a total of 61,500 ED presentations and 7,089 admissions for paediatric asthma. Figure 1 shows the mean daily ED visits per million stratified by age (2–5 years, 6–12 years, and 13–17 years) and sex. The mean daily ED visits remained between 5 and 10 per million per year in children from 2 to 12-years-old, and below 5 per million per year in children from 13 to 17-years-old before the pandemic. There was a sharp decline in ED visits in 2020 followed by a notable rebound in 2021, particularly in the youngest age group (2–5 years). Males under 12 years of age consistently showed a higher incidence of ED visits than females. The youngest age group showed the highest incidence of ED visits and the oldest age group had the lowest incidence. The overall trend shows a return to pre-pandemic levels by the late pandemic, in 2022-23 (Fig S1).

## Hospital admission rates

**Fig. 2:**
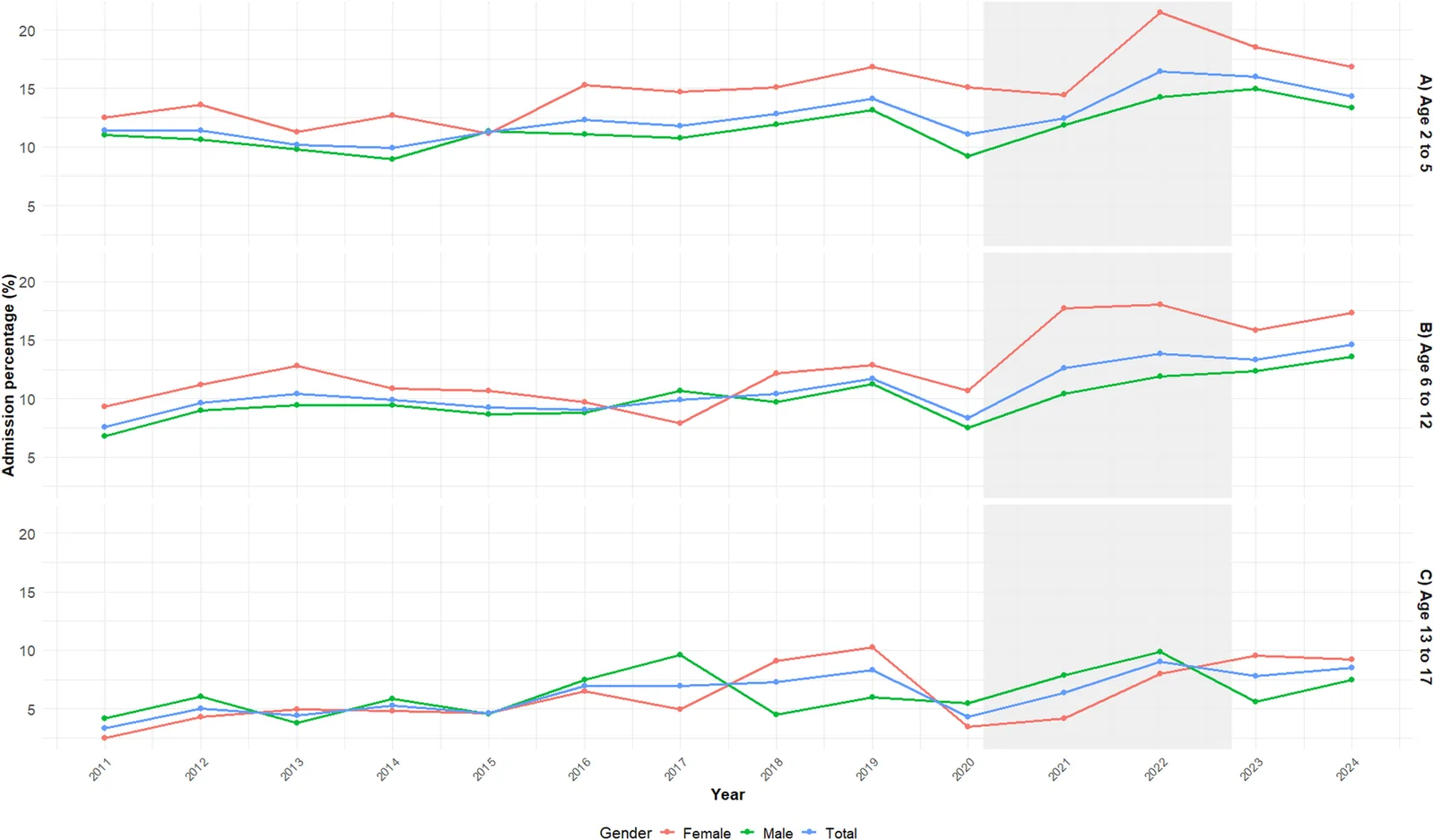
Admission percentage categorized by sex across three age groups: **A** 2 to 5 years, **B** 6 to 12 years, and **C** 13 to 17 years. The grey shaded area represents the COVID-19 pandemic (March 2020 to September 2022)

Figure 2 shows the admission percentage from 2011 to 24 stratified by age and sex. The admission percentage reflects the number of children hospitalized for asthma relative to the number of children presenting to the ED for asthma exacerbations. The admission percentage generally increased in all groups over this time. This was transiently interrupted in 2020, at the beginning of the pandemic. The overall trends mirror the male trends, given most children presenting to the ED with asthma were male. The average unstratified admission percentage from 2011 to 2019 was 10.3%, while in 2024 it increased to 14.1%. By 2024, the admissions percentage was similar to the pre-pandemic period in adolescents, and greater than the pre-pandemic period in children aged 2–12 years.

### ED Visits via emergency medical services (EMS)

**Fig. 3:**
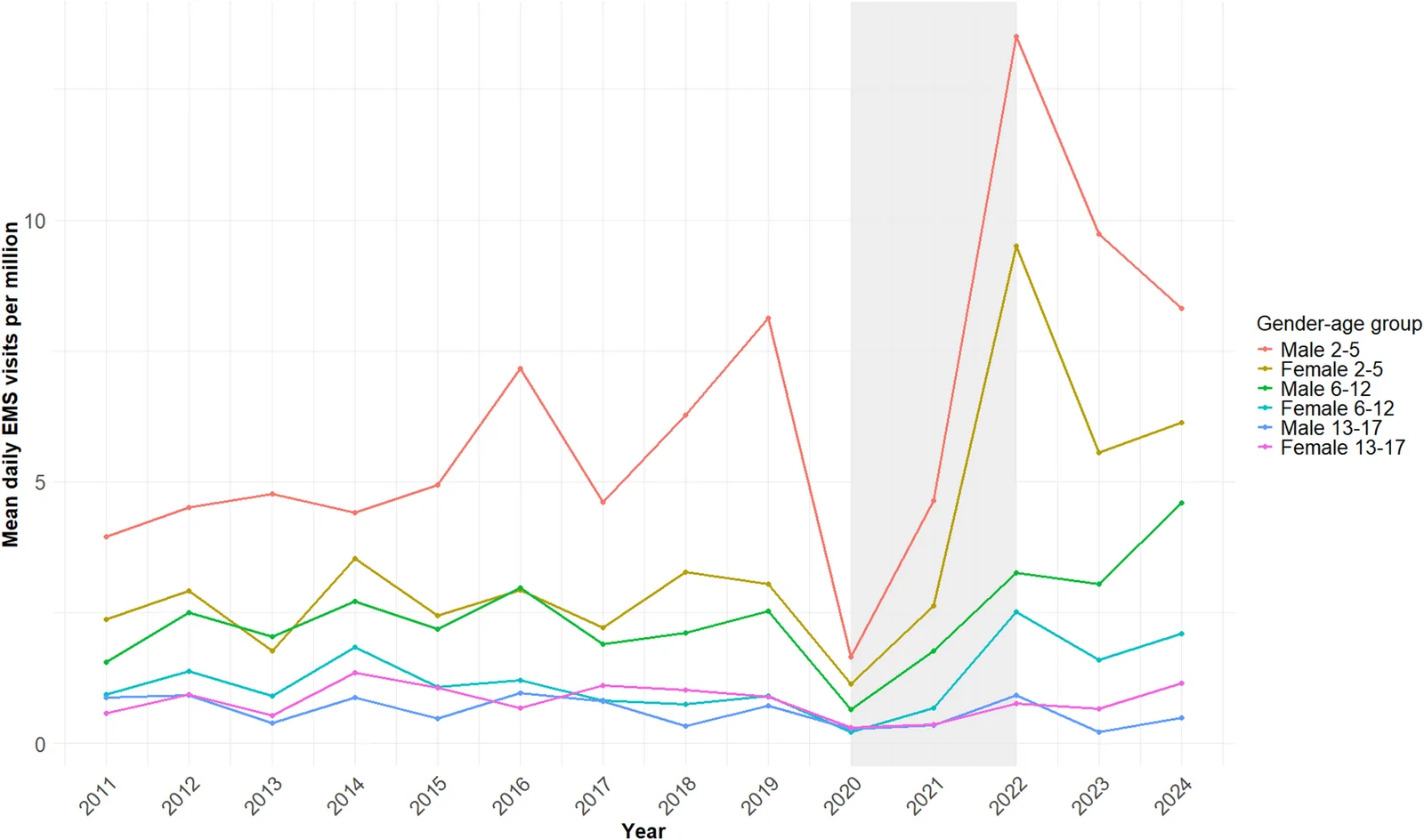
Mean daily ED visits via EMS per year stratified by age and sex (2011 to 2024)

Figure 3 shows the mean daily ED visits for asthma that arrived by EMS per year stratified by age and sex. Males aged 2–5 had the highest EMS utilisation for asthma in all years. There was a sharp drop in EMS utilisation in 2020, followed by a rebound in 2021. EMS utilisation remains at levels similar to or higher than pre-pandemic levels in children from 2 to 12 years old. The pre-existing trend to lower EMS utilisation has continued in adolescents in the post-pandemic years.

The range of mean daily ED visits per million among adolescents (ages > 12) across the study period was 0.22 to 1.36. Among males aged 2–5 years, the highest mean daily ED visit occurred in 2022, reaching 13.5 visits per million. When comparing the average daily ED visit during the pre-pandemic period (2011–2019) to those in 2024, we observed notable increases across all younger age groups. Specifically, mean daily visits in 2024 were 1.53× higher for males aged 2–5, 2.26× higher for females aged 2–5, 2.02× higher for males aged 6–12, and 1.93× higher for females aged 6–12.

## Seasonal effects on ED visits

**Fig. 4:**
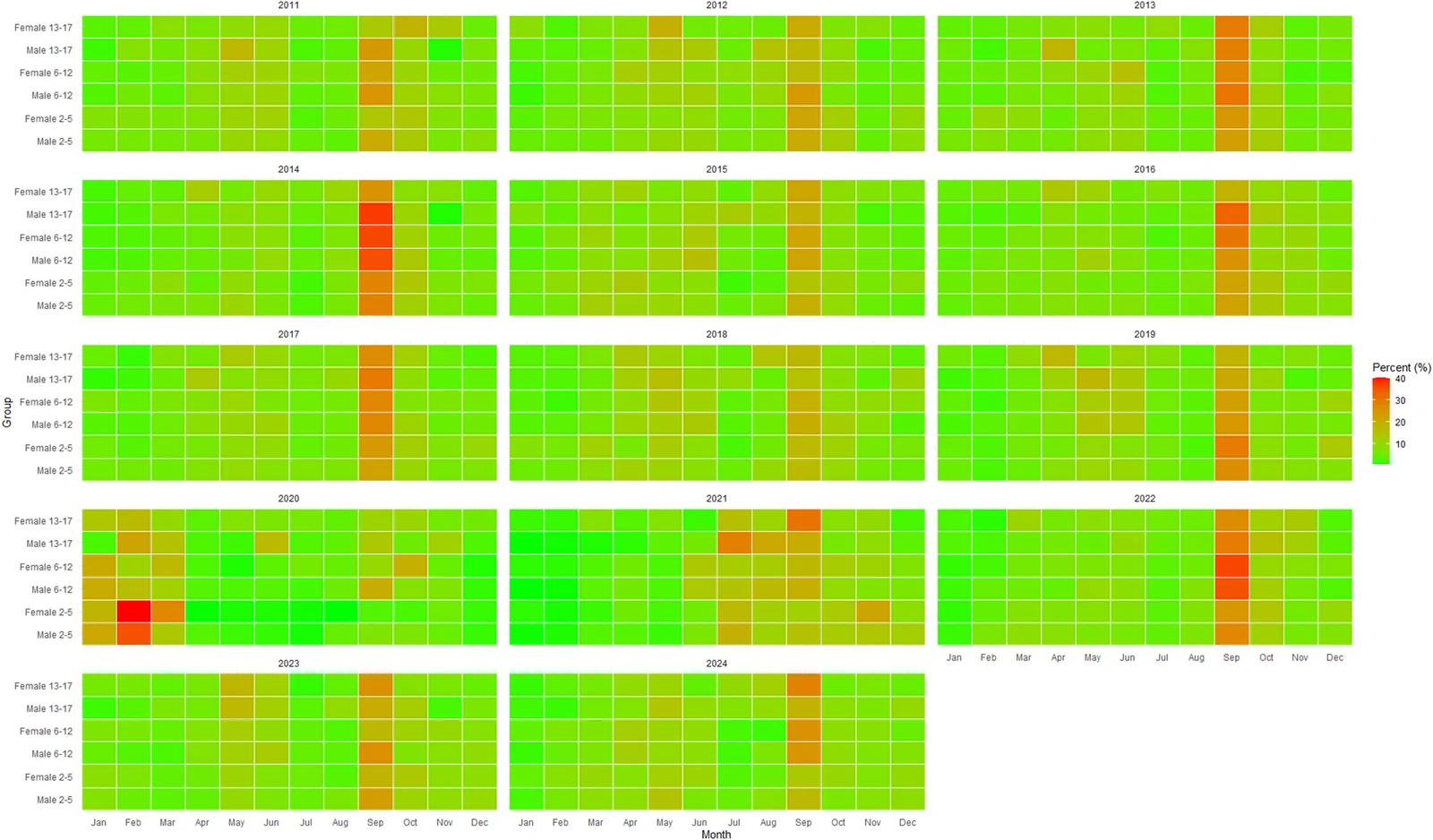
Monthly proportion of ED visits by age and sex from 2011 to 2024. The colours on the heat map represent the proportion of paediatric ED visits that month relative to the calendar year. The colours are not directly comparable year-to-year

Figure 4 shows the proportion of the year’s total ED visits per month. Pre-pandemic (2011-19), ED visits were relatively stable most of the year, averaging approximately 8.3% per month, with a consistent “September spike”, during which 17.8 to 31.9% of annual ED visits occurred. In the early pandemic (2020–2021), the September spike was greatly diminished, in 2020, only 11.2% of visits occurred in September, and in 2021, just 16.4%. Normal seasonality in ED visits started to return in 2021, and the September spike returned clearly by 2022, with 29.5% of annual visits occurring in that month, the highest in the post-pandemic period.

## Trends in paediatric ED visits by CTAS

**Fig. 5:**
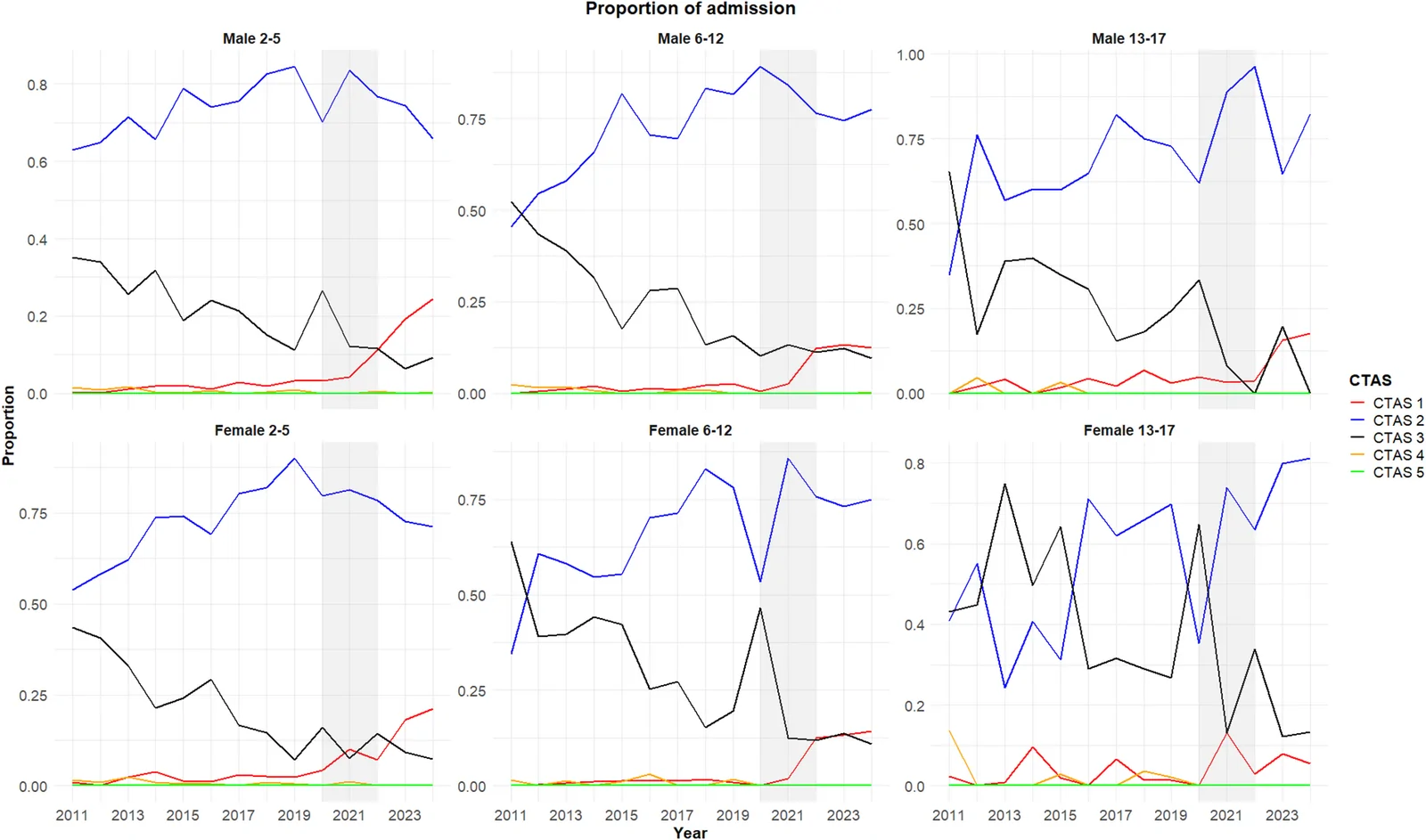
Trends in admission proportion by CTAS, age, and sex from 2011 to 2024. The admission proportion represents the number of children in a CTAS category relative to the total number of children presenting to the ED with asthma per year

Figure 5 presents admissions categorized by CTAS stratified by age and sex over time. There was a trend to increasing CTAS 2 and lower CTAS 3 cases prior to the pandemic, meaning the acuity of children with asthma in the ED was increasing. For example, for males aged 6–12, the proportion of CTAS 2 cases increased from 0.45 to 0.82 from 2011 to 2019, while the proportion of CTAS 3 cases decreased from 0.52 to 0.16 in the same period.

This trend was interrupted in 2020, when CTAS 2 cases dipped and CTAS 3 cases spiked, suggesting asthma exacerbations were milder in that year. By 2021, CTAS 3 cases had declined to pre-pandemic levels or lower. The highest acuity cases, CTAS 1 and CTAS 2, cases began to rise, and this rise was sustained in the post-pandemic years. In females aged 2–5, from 2019 to 2024 the proportion of CTAS 1 cases increased from 0.02 to 0.21, while the proportion of CTAS 2 cases decreased from 0.90 to 0.71. In males aged 2–5, the proportion of CTAS 1 cases increased from 0.03 to 0.24, and the proportion of CTAS 2 cases decreased from 0.85 to 0.66. There were very few of the lowest-acuity CTAS 4 and 5 cases throughout the study period.

### Interrupted time-series analyses

**Fig. 6:**
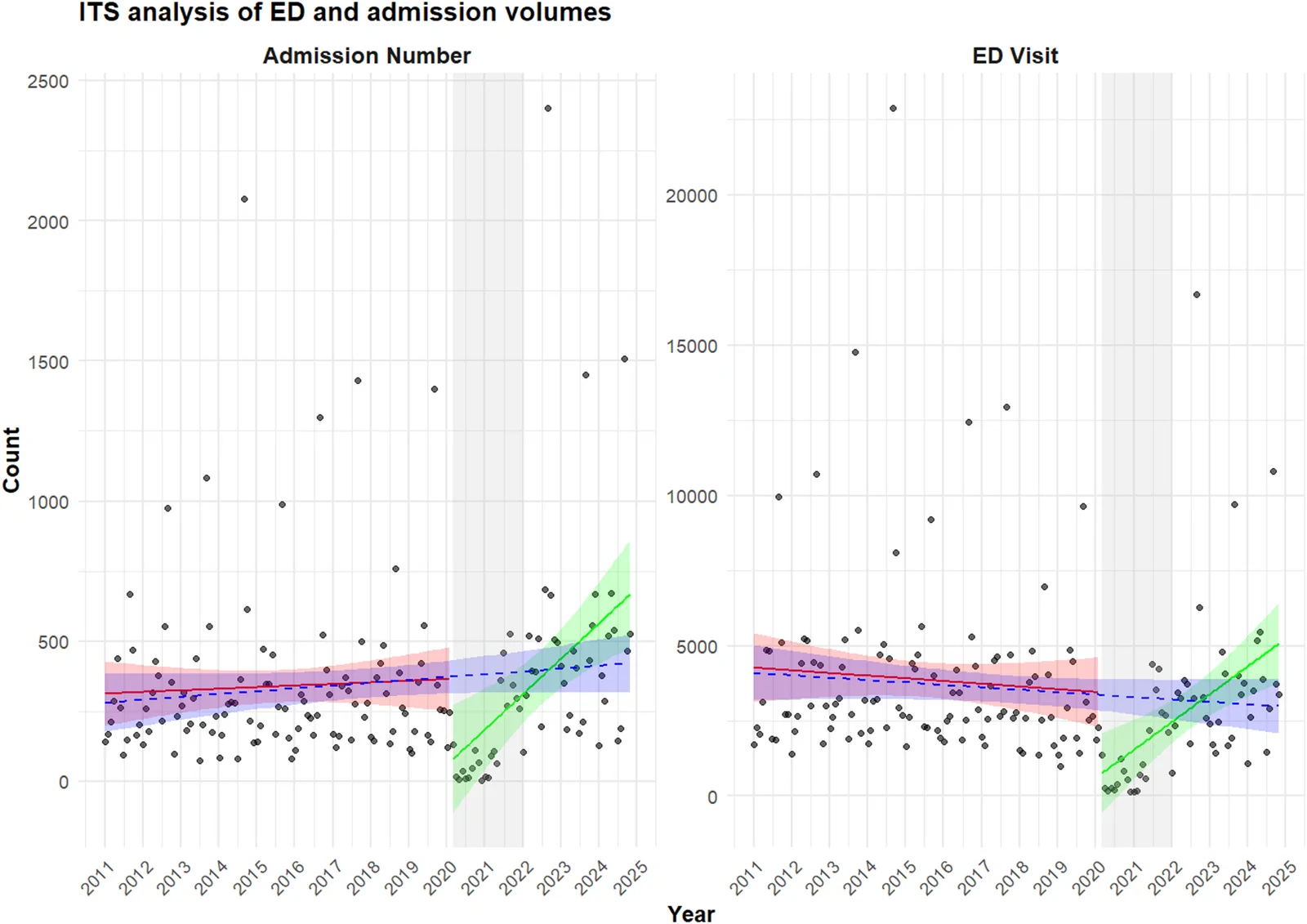
Interrupted time series of ED Visits and admission number before and after the COVID-19 pandemic

Figure 6 presents the results of the ITS analyses for ED visits and hospital admissions over the study period. Prior to the COVID-19 pandemic, outcome rates showed a stable or modest trend over time. At the onset of the pandemic (March 2020), there was a significant immediate decline in ED visits and hospital admissions, indicating a negative level change. In the post-pandemic period, a gradual increase in rates was observed, reflecting a positive change in slope, with utilization partially or fully returning toward pre-pandemic levels.

Stratified analyses demonstrated important differences by acuity and age (Fig S2). Reductions were most pronounced among lower-acuity visits (CTAS 4–5), whereas higher-acuity visits (CTAS 1–2) were less affected and showed relatively stable or increasing trends over time. Age-specific analyses indicated that younger individuals experienced larger initial declines and faster recovery, while older age groups exhibited more stable patterns.

## Discussion

We conducted a retrospective study of all ED visits for childhood asthma in Alberta from 2011 to 24. Prior to the COVID-19 pandemic, the number of ED visits was stable, at 5–10 per million per year in children from 2 to 12-years-old, and under 5 per million per year in adolescents. The pre-existing trend of increasing acuity accelerated in the late pandemic, with increasing CTAS 1 and 2 cases, fewer CTAS 3 cases, and this was associated with increasing rates of hospital admission. This increased acuity continued in the post-pandemic period until the end of 2024. The trend to increased acuity is corroborated by EMS data. There was a sharp reduction in ED visits requiring EMS in the early pandemic, in 2020. The following year, there was a rebound in paediatric asthma requiring EMS and EMS utilisation has remained similar or higher than pre-pandemic levels in the pre-adolescent age groups.

This work builds on prior studies, which found a sharp reduction in paediatric hospitalizations for acute respiratory diseases in Alberta in 2020, followed by a return to pre-pandemic levels by 2023-24 [4]. We found a similar decline in paediatric ED presentations for asthma in 2020 and a return to pre-pandemic levels by about 2022. Both prior studies found the pandemic disrupted the seasonality of respiratory diseases in 2020, particularly the September spike in asthma ED presentations, which is a well-documented phenomenon [10, 11]. Our study goes further by showing the acuity of paediatric asthma exacerbations in the ED has been increasing for years, and this trend accelerated in the late pandemic and thereafter. It appears that children with asthma are not presenting to the ED unless they have particularly severe symptoms, and this long-standing behavioural trend increased in 2021-22.

Interestingly, Lukac et al., demonstrated that paediatric intensive care unit admissions for acute respiratory diseases and asthma were similar in the pre-pandemic and post-pandemic periods [6]. Our data would suggest that children with CTAS 2 or 3 exacerbations who would previously have gone to intensive care are now being managed in the ED or regular ward.

The data here are consistent with our prior study of ED visits for asthma in adults from 2010 to 22. Although ED visits remained relatively stable during the pre-pandemic period, the acuity of exacerbations worsened, including CTAS 1 cases. We posited that the prevalence of poorly controlled asthma in the community was increasing, but patients were reluctant to come to the ED due to overcrowding. Asthma control in the community may have worsened due to insufficient prescription of asthma controller medications, increasing rates of obesity, climate change, forest fires, e-cigarette use, marijuana, and a primary health care system under strain. This reasoning would also apply to the paediatric population. The paediatric study extends our observations to the post-pandemic period and show the trends to higher acuity accelerated in the late pandemic and post-pandemic years.

The worsening of paediatric asthma acuity in the post-pandemic years is a new finding. COVID-19 itself may alter the immune system and predispose individuals to allergic diseases and increase disease severity, which could partly account for this observation [12, 13]. During the late pandemic, there was a decline in physician visits, accompanied by a reduction in inhaled corticosteroid (ICS) and short-acting beta agonist prescriptions filled by Canadian children [14]. This likely exacerbated a pre-existing decade-long decline in asthma controller prescriptions among children in Alberta [15]. Despite the resolution of lockdowns, continued avoidance of the ED is a legacy of the pandemic. This may be due to long wait times, fear of contagion, or a cultural shift not to go to the ED except *in extremis* [16, 17]. As such, EDs must be prepared to manage a higher number of children with CTAS 1 asthma, including those needing high-flow nasal cannula or mechanical ventilation.

Our finding that boys had significantly more asthma-related ED visits than girls aligns with previous studies, which identify male sex as a strong predictor of persistent asthma and increased asthma severity in childhood [18, 19]. A population-based cohort study in Ontario found that boys were more likely to have persistent asthma than girls (52.9% vs. 49.1%, *p* < 0.001) and that children with early asthma hospitalizations had a 3-fold increased risk of persistent asthma [20]. Our ITS analysis showed a marked decline in ED visits and hospital admissions at the onset of the COVID-19 pandemic, followed by a gradual recovery over time.

Limitations of this study included the lack of other demographic and co-morbidity factors in our analysis, which may influence asthma severity and health care utilisation patterns. Factors such as socioeconomic status, ethnicity, household environment, and co-existing respiratory conditions were not accounted for. We excluded children from 0 to 2-years-old due to the difficulty of distinguishing asthma from other respiratory diseases in this age group. Our reliance on administrative data introduces potential coding errors and biases. There was no control group to determine if changes in acuity and EMS use were broad changes in healthcare delivery in Alberta or specific to asthma.

## Conclusions

The acuity of asthma exacerbations in young children has worsened in the post-pandemic period, in Alberta, Canada, resulting in higher EMS use and higher rates of hospital admissions. Further research with patient level data will be helpful to assess whether patient-related or health system factors are the dominant drivers of these changes. Interventions to improve outpatient care that leads to greater controller adherence and asthma action plan use may be cost effective approaches to combat this worrisome trend.

## Supplementary Information

Below is the link to the electronic supplementary material.

Supplementary Material 1

## Data Availability

Data are publicly available from the Government of Alberta Interactive Health Data Application (http://www.ahw.gov.ab.ca/IHDA_Retrieval/selectCategory.do). All relevant summary data are presented in the manuscript.
